# 

**Published:** 1892-05

**Authors:** 


### PRELIMINARY PROGRAMME OF THE AMERICAN ACADEMY OF MEDICINE.

---

The following topics are promised for discussion at the seventeenth annual meeting of the American Academy of Medicine at the Cadillac Hotel, Detroit, Mich., on Saturday, June 4, and Monday, June 6, 1892.

1. "Essentials and Non-essentials in Medical Education," the address of the retiring President, Dr. P. S. Conner, of Cincinnati.

2. "The Value of the General Preparatory Training Afforded by the College as Compared with the Special Preparatory Work Suggested by the Medical School in the Preliminary Education of the Physician," a paper by Dr. T. F. Moses, of Urbana, O.

3. "Does a Classical Course Enable a Student to Shorten the Period of Professional Study?" a paper by Dr. V. C. Vaughan, of Ann Aabor, Mich.

4. "The Value of a Collegiate Degree an Evidence of Fitness for the Study of Medicine," a paper by Dr. L. H. Mettler, of Chicago.

5. "The Value of Academical Training Preparatory to the Study of Medicine;" a symposium, by Drs. H. B. Allyn, of Philadelphia, W. D. Bidwell, of Washington, and Elbert Wing of Chicago.

6. "The Newer Medical Education in the United States;" a symposium by Drs. W. J. Herdman, of Ann Arbor, Charles Jewett, of Brooklyn, and Elbert Wing, of Chicago.

7. "A Paper on Some Phase of the State Supervision of the Practice of Medicine," by Perry H. Millard, of St. Paul.

Some other papers are partially promised, and the usual reports may be expected from the committees.

Members of the profession are cordially invited to be present at the session of the Academy.

